# Comparison of Echocardiographic and Electrocardiographic Mapping for Cardiac Resynchronisation Therapy Optimisation

**DOI:** 10.1155/2019/4351693

**Published:** 2019-02-21

**Authors:** Helder Pereira, Tom A. Jackson, Simon Claridge, Jonathan M. Behar, Cheng Yao, Benjamin Sieniewicz, Justin Gould, Bradley Porter, Baldeep Sidhu, Jaswinder Gill, Steven Niederer, Christopher A. Rinaldi

**Affiliations:** ^1^Division of Imaging Sciences and Biomedical Engineering, King's College London, London, UK; ^2^Cardiac Rhythm Management Service, St George's University Hospitals NHS Foundation Trust, London, UK; ^3^Cardiovascular Department, Guy's and St Thomas' NHS Foundation Trust, London, UK; ^4^Medtronic Ltd, UK

## Abstract

**Study hypothesis:**

We sought to investigate the association between echocardiographic optimisation and ventricular activation time in cardiac resynchronisation therapy (CRT) patients, obtained through the use of electrocardiographic mapping (ECM). We hypothesised that echocardiographic optimisation of the pacing delay between the atrial and ventricular leads—atrioventricular delay (AVD)—and the delay between ventricular leads—interventricular pacing interval (VVD)—would correlate with reductions in ventricular activation time.

**Background:**

Optimisation of AVD and VVD may improve CRT patient outcome. Optimal delays are currently set based on echocardiographic indices; however, acute studies have found that reductions in bulk ventricular activation time correlate with improvements in acute haemodynamic performance.

**Materials and methods:**

Twenty-one patients with established CRT criteria were recruited. After implantation, patients underwent echo-guided optimisation of the AVD and VVD. During this procedure, the participants also underwent noninvasive ECM. ECM maps were constructed for each AVD and VVD. ECM maps were analysed offline. Total ventricular activation time (TVaT) and a ventricular activation time index (VaT_10-90_) were calculated to identify the optimal AVD and VVD timings that gave the minimal TVaT and VaT_10-90_ values. We correlated cardiac output with these electrical timings.

**Results:**

Echocardiographic programming optimisation was not associated with the greatest reductions in biventricular activation time (VaT_10-90_ and TVaT). Instead, bulk activation times were reduced by a further 20% when optimised with ECM. A significant inverse correlation was identified between reductions in bulk ventricular activation time and improvements in LVOT VTI (*p* < 0.001), suggesting that improved ventricular haemodynamics are a sequelae of more rapid ventricular activation.

**Conclusions:**

EAM-guided programming optimisation may achieve superior fusion of activation wave fronts leading to improvements in CRT response.

## 1. Introduction

Cardiac resynchronisation therapy (CRT) is recommended for patients with systolic heart failure, prolonged QRS duration, and left bundle branch block [1, 2]. Despite the fact that CRT has been available for more than 20 years, up to 30% of patients fail to respond to this therapy [3]. Left ventricular (LV) pacing alone has been proposed as an alternative to biventricular pacing, allowing for simpler systems that avoid the complication of right ventricular pacing [4]. However, some features of cardiac remodelling respond better to biventricular pacing compared with LV pacing, suggesting that optimisation of biventricular pacing should be pursued in CRT [5, 6]. One approach designed to improve CRT response is optimisation of the pacing delay between the atrial and ventricular leads (atrioventricular delay or AVD), and the delay between the ventricular leads (interventricular pacing interval or VVD) for each individual patient [7]. While there are multiple strategies for AVD and VVD optimisation, there is no clear “gold standard” and existing guidelines do not provide recommendations [7]. As a consequence, different protocols are used that either consider echocardiographic parameters or use electrograms to determine the optimal device timings [8].

CRT aims at eliminating the dyssynchrony, which results from bundle branch block activation, by reducing the left ventricular activation time (LVaT) and restoring the mechanoenergetic efficiency of the heart. Rapid LV activation is preferred and is associated with improvements in functional class and symptoms [9, 10]. Sohal et al. [11] reported a difference in LVaT between responders and nonresponders to CRT, with responders exhibiting greater activation homogeneity, measured using the delay between the 10th and 90th percentiles of LVaT (LVaT_10-90_ Index). The cumulative rate of LV activation appears critical, a finding consistent with previous modelling studies [11, 12].

CRT programming aims at resynchronising the electrical activity to ensure the optimal fusion of all activation wave fronts: intrinsic right ventricular depolarisation, RV paced activation, and LV depolarisation [13]. Patients with partial fusion of their intrinsic depolarisation with LV pacing have been found to have greater LV reverse remodelling and haemodynamic response [14]. Furthermore, the use of electrocardiographic indices to optimise AVD to achieve optimal activation wavefront fusion is associated with significant improvements in acute haemodynamic response (AHR) [15]. Another development capable of improving AHR is multipolar pacing (MPP), where stimulation is delivered from multiple poles along the LV lead, allowing the avoidance of pacing in and around scar. This technique has been associated with improvements in CRT response [16].

The close relationship between activation wave fusion and AHR suggests that the use of electrical indices for CRT optimisation would be beneficial. The recent availability of noninvasive electrocardiographic mapping (ECM) means detailed, patient-specific biventricular activation can now be calculated noninvasively [17, 18].

## 2. Hypothesis and Study Aim

We sought to investigate the association between echocardiographic optimisation and ventricular activation time, obtained through the use of ECM. We hypothesised that echocardiographic optimisation of AVD and VVD would correlate with reductions ventricular activation time.

## 3. Materials and Methods

We undertook a prospective study recruiting consecutive heart failure (HF) patients indicated for CRT-pacemaker (CRT-P) or CRT-defibrillator (CRT-D) at St Thomas' Hospital, London. The study conformed to the principles outlined in the Declaration of Helsinki on research in human subjects. All patients gave written informed consent to participate in the study, which was approved by the Research Ethics Committee (ClinicalTrials.gov Identifier: NCT01831518). We aimed at recruiting 20 patients within 18 months, and the first patient was recruited in September 2014 and the last patient in November 2015. In total, 21 patients were selected on the basis of fulfilling the criteria for CRT implantation: NYHA Class II–IV; echocardiographic Left Ventricular Ejection Fraction (LVEF) < 35%; QRS duration > 120 ms (independently of the QRS morphology); and optimal medical therapy (OMT) for heart failure. The aetiology of heart failure was classified as ischaemic if there was substantial coronary artery disease or history of myocardial infarction or revascularisation and as nonischaemic if none of these were present. Intraventricular conduction disturbances were defined according to AHA/ACCF/HRS Recommendations for the Standardisation and Interpretation of the Electrocardiogram [19]. 12-lead ECGs were acquired with a GE Mac 5000 ECG system (General Electric-Vingmed, Milwaukee, WI) using standard American Heart Association- (AHA-) recommended filter settings at a sweep rate of 25 mm/s and a gain of 10 mm/mV. Echocardiography was performed using an IE33 or EPIC model scanner (Philips Healthcare, Best, The Netherlands).

### 3.1. CRT Implantation

Implantation was performed via the cephalic, axillary, or subclavian veins. The RV lead was implanted at the RV apex or high septum at the discretion of the implanting physician, and the right atrial lead was placed at the right atrial appendage. The LV lead was preferentially placed in the lateral or posterolateral vein tributary of the coronary sinus. In case of technical difficulties, unacceptable pacing thresholds or phrenic nerve stimulation, an alternative location was chosen in the anterolateral, posterior, or anterior regions.

### 3.2. Echocardiographic Optimisation

Echocardiographic optimisation of the AVD and VVD was performed the day after implantation, with the exception of patients with atrial fibrillation who had only their VVD but not their AVD echocardiographically optimised. Varying AV intervals were progressively applied (from 60 ms to 200 ms in 20 ms increments), and the echocardiographic optimal AVD was calculated using an iterative method based on the maximal separation of E and A waves recorded by pulsed-wave Doppler of diastolic mitral inflow and the maximal mitral velocity-time integral (VTI), as previously described [7, 20]. The AVD with distinct E- and A-waves, yielding the maximal atrial contribution to ventricular filling and minimal mitral regurgitation, was considered the optimal AVD. VVD optimisation was performed following AVD optimisation, starting with simultaneous RV and LV pacing. Varying VVD was applied by progressively increasing LV preexcitation in increments of 15, 20, 30, and 40 ms, and then increasing RV preexcitation in increments of 20 and 40 ms. The optimal VVD was defined as the delay producing the maximal LVOT VTI, which represents the maximal LV stroke volume (a reproducible measure of global LV function that has proven to be useful for improving the response to CRT) [21]. The effects of each applied AVD and VVD setting on mitral and LVOT VTI were assessed after 10 consecutive beats in order to minimise the effects of beat-to-beat variability in optimisation measures, which have been shown to be substantially and potentially limiting in research settings [22]. It should be noted that the LVOT VTI method was preferred to other haemodynamic outcome measures (e.g., dp/dtmax) as this is a feasible, noninvasive, reproducible, and direct measure of global LV function, comparable to other measures [23].

### 3.3. Electrocardiographic Mapping

**Figure 1:**
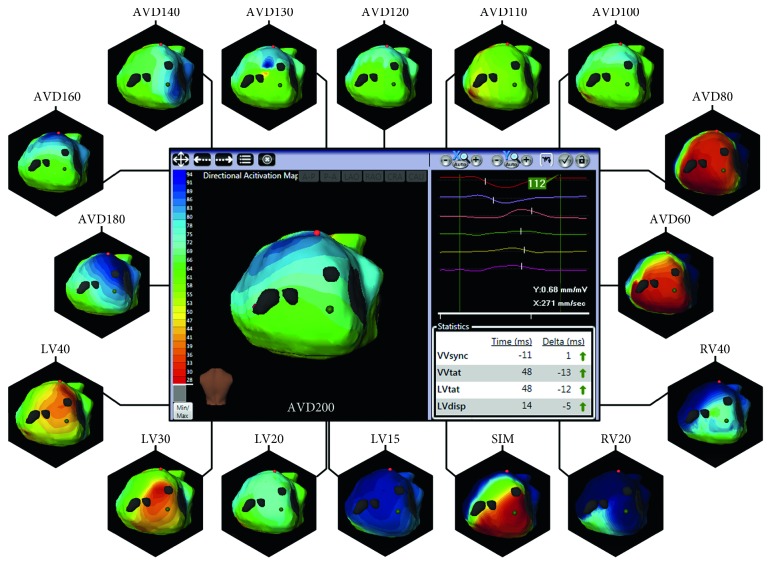
Example of 3D epicardial isochrone maps obtained for patient 7 during CRT optimisation where the optimal atrioventricular delay (AVD) and VV were identified through echo-guided optimisation.

During AVD and VVD optimisation, patients underwent ECM using a CardioInsight ECSYNC system (CardioInsight Technologies Inc., Cleveland, OH, USA) to noninvasively record biventricular epicardial ventricular electrograms and construct 3D isochrone and isopotential activation maps. The key component of the ECM system is a vest embedded with 252 electrodes that is fitted to the patient's torso. ECM maps were constructed on a beat-by-beat basis for the different AVD and VVD tested. After optimisation and acquisition of vest electrograms under each configuration, the participants, with the vest still in position, underwent a thoracic computed tomographic (CT) scan to determine the precise anatomic relation between the cardiac geometry and the torso electrodes, which was used to reconstruct approximately 1500 unipolar electrograms on the epicardial surface of the heart. Based on each data set obtained with the ECSYNC, an activation map of both ventricles was generated offline by animating the activation waveform on the patient-specific CT-derived epicardial surface. Ventricular activation times were calculated from the onset of the QRS to the maximal negative slope of each electrogram and combined for the construction of 3D epicardial isochrone maps. The propagation of depolarisation was evident from the 3D epicardial isochrone maps (Figure 1). Subsequently, extraction of specific raw data from epicardial maps obtained at baseline and in each AVD and VVD assessed permitted the calculation of total ventricular activation time (TVaT) and ventricular activation time 10-90 index (VaT10-90) with custom-developed MATLAB code (MathWorks, Natick, MA, USA) as previously described by Pereira et al. [24]. TVaT is a measure of the total time required for both ventricles to activate, and VaT_10-90_ is the time delay between the 10th and 90th percentiles of activation.

### 3.4. Statistical Analysis

Statistical analyses were performed using PASW Statistics 21 (SPSS Inc., Chicago, IL). Changes in ventricular activation times were compared using the Mann–Whitney *U* test, ANOVA, and Kruskal–Wallis test. Post hoc comparisons were performed using Tukey's HSD. Correlations were assessed by the Pearson correlation test. *p* values less than 0.05 were deemed statistically significant.

## 4. Results and Discussion

The characteristics of the 21 patients are shown in Table 1. The mean age was 69 ± 12 years. Patients were predominantly male, and most had an ischaemic aetiology (62%). The mean LVEF was 27 ± 10%, and the mean QRS duration was 162 ± 21 ms. Fifteen patients (71%) had QRS >150 ms, and 15 (71%) had left bundle branch block. Baseline values are shown in Table 2.

### 4.1. AV Optimisation and Electrical Timing

**Figure 2:**
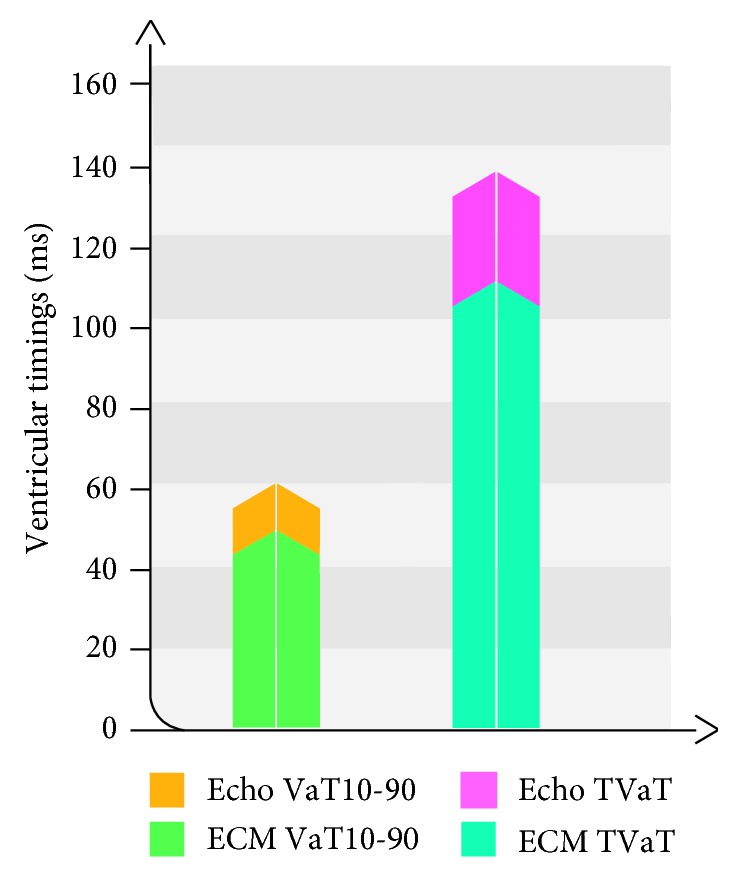
Differences in ventricular activation timings in AVD optimisation by echocardiographic methods and ECM. AVD, atrioventricular delay; echo, echocardiographic mapping; echo TVaT, mean of the TVaT obtained based on the optimal AVD obtained through echocardiographic methods; Echo VaT10-90, mean of the VaT_10-90_ index obtained based on the optimal AVD obtained through echocardiographic methods; ECM, electrocardiographic mapping; ECM VaT10-90, mean of the shortest VaT_10-90_ index obtained for the AVD tested; ECM TVaT, mean of the shortest TVaT obtained for the AVD tested.

The effects of varying AVD on ventricular activation time are shown in Table 3. There was no significant difference in TVaT (*p*=0.98) or VaT_10-90_ index (*p*=0.701) between the different AVD values tested across the cohort, suggesting that no single AVD was optimal for electrically synchronizing all patients. The shortest VaT_10-90_ index was seen with AVD 100 ms (62 ± 20 ms), and longer VaT_10-90_ index values were observed with longer AVDs, especially with AVD 200 ms (VaT_10-90_ index 81 ± 21 ms). In contrast, the shortest AVD tested (AVD 60 ms) gave the longest TVaT (147 ± 26). The optimal AVD found with echocardiographic optimisation did not correspond to the shortest ventricular times observed. The average VaT_10-90_ and TVaT values were 21% and 20% lower, respectively, than the optimal AVD found through the iterative method (Figure 2). Whilst these findings failed to achieve statistical significance (*p*=0.368), this is in part explained by the potential for large variability in beat-to-beat and test-retest measurement of LVOT VTI [22].

**Figure 3:**
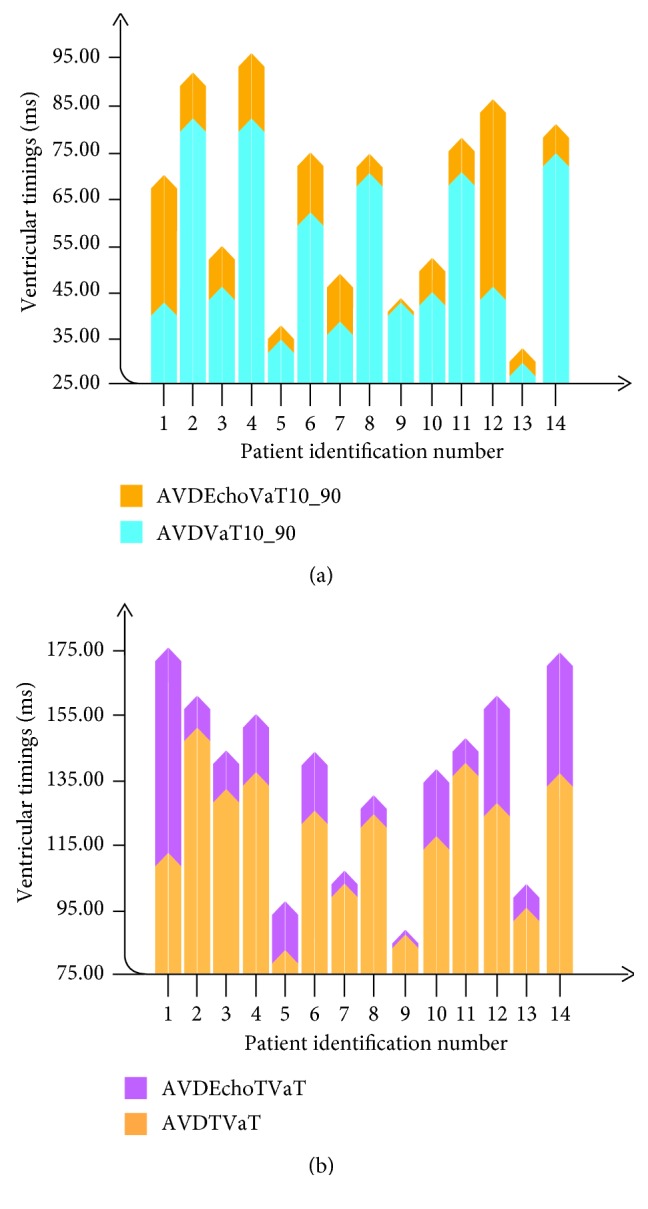
Activation timings for the optimal AVD obtained by the iterative method versus the shortest ventricular activations obtained for each patient. AVD, atrioventricular delay; AVDEchoTVaT, TVaT obtained based on the optimal AVD obtained through echocardiographic methods; AVDEchoVaT10-90, VaT_10-90_ index obtained based on the optimal AVD obtained through echocardiographic methods; AVDVaT10-90, shortest VaT_10-90_ index obtained for the AVD tested; AVDTVaT, shortest TVaT obtained for the AVD tested; XX patient identification number.

**Figure 4:**
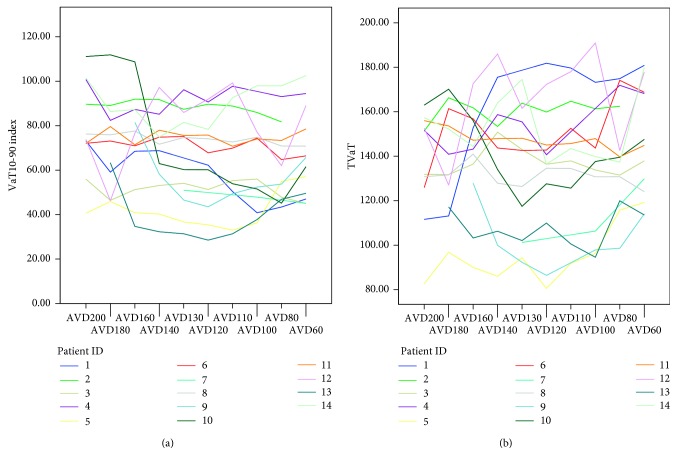
VaT_10-90_ and TVaT values in each patient versus the AVD setting. AVDXXX, atrioventricular delay at XXX milliseconds; TVaT, total ventricular activation time; VaT10-90 index, ventricular activation time between percentile 10 and 90 obtained.

Echocardiographic CRT optimisation consistently failed to achieve the greatest reduction in ventricular activation (Figure 3). Two groups of patients were identified: those with clear optimal value that was well distinguished within the evaluated AVD's range (60%) and those in which AVD settings had very limited effect on TVaT or VaT_10-90_ index (40%) (Figure 4).

### 4.2. VVD Optimisation and Electrical Timings

**Figure 5:**
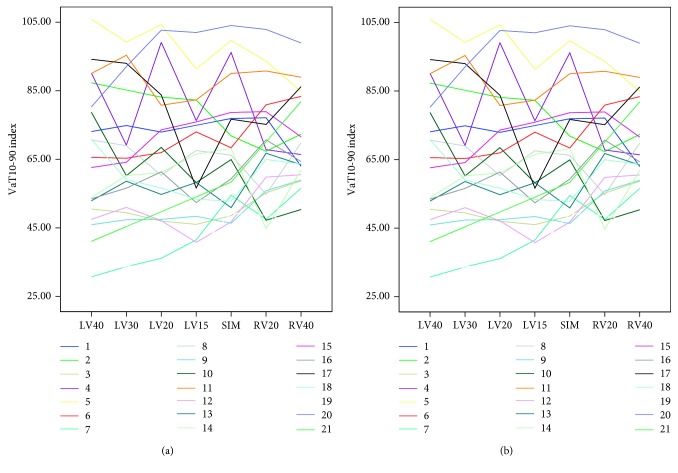
VaT_10-90_ and TVaT values in each patient according to the VVD setting. LVx, intraventricular pacing interval with the left ventricle paced first by x milliseconds; RVx, intraventricular pacing interval with the right ventricle paced first by x milliseconds; SIM, intraventricular pacing interval with the right and left ventricles paced simultaneously; TVaT, total ventricular activation time; VaT_10-90_, ventricular activation time_10-90_.

**Figure 6:**
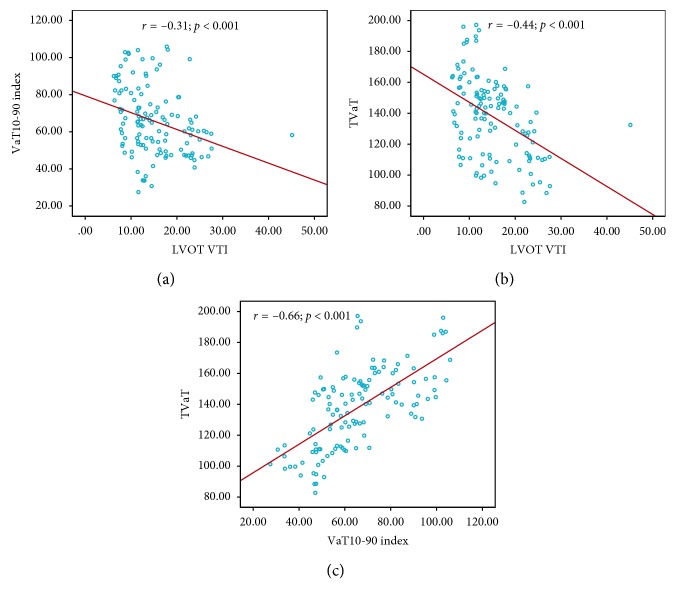
Correlations among LVOT VTI, TVaT, and VaT_10-90._ LVOT VTI, left ventricular outflow tract velocity time integral; TVaT, total ventricular activation time; VaT_10-90_, ventricular activation time_10-90_. Pearson correlation; *p* < 0.001 for LVOT VTI/TVaT and LVOT VTI/VaT_10-90_.

The effects of each applied VVD on ventricular activation times and LVOT VTI are shown in Table 4. LVOT VTI values were higher when LV was programmed to be paced before RV, by either 15 ms or 30 ms (LV15 and LV30), and were associated with the shortest values for the VaT_10-90_ index. LV15 appeared to offer the highest LVOT VTI and the shortest VaT_10-90_ index and TVaT. No single VVD achieved significant reductions in ventricular activation time when plotted for each patient (Figure 5). A negative correlation between LVOT VTI and VaT_10-90_ index (*r*  =  −0.31; *p* < 0.001) and between LVOT VTI and TVaT (*r*  =  −0.44; *p* < 0.001) (Figure 6) was observed.

### 4.3. Findings and Comparison with Previous Studies

We assessed if the optimal parameters obtained through echocardiographic CRT optimisation rendered similar AVD and VVD timings as assessed by ECM. The main findings of this study were as follows:

1. Echocardiographic programming optimisation was not associated with the greatest reductions in biventricular activation time (VaT_10-90_ and TVaT). Instead, bulk activation times were reduced by a further 20% when optimised with ECM.
2. A significant inverse correlation was identified between reductions in bulk ventricular activation time and improvements in LVOT VTI (*p* < 0.001), suggesting that improved ventricular haemodynamics are a sequelae of more rapid ventricular activation.

In keeping with previous studies, we identified that echocardiographic optimisation and ECM optimisation were patient-specific. However, ventricular activation was consistently more rapid when optimised via ECM than when echocardiographic optimisation was performed. These findings appear to suggest that programming changes which improve mitral inflow and left ventricular filling do not necessarily achieve a reduction in total ventricular activation time raising the question as to whether AVD should be set to achieve optimal filling, optimal electrical synchrony, or potentially a combination of the two.

LVOT VTI is widely accepted as an echocardiographic parameter positively correlated with both stroke volume and cardiac output [25]. Previous work has highlighted the haemodynamic benefits of minimising ventricular activation time (Vatasescu et al.) [13]. Our finding of a significant inverse correlation between increasing LVOT VTI and decreasing ventricular activation time, measured using noninvasive ECM, suggests a future role for electrical optimisation using this approach, when looking to maximise cardiac output.

### 4.4. Clinical Relevance

Our findings suggest that when looking to optimise CRT programming, a strategy of aiming at minimising ventricular activation is associated with significant improvements in LVOT VTI. In addition, this approach is associated with a greater degree of electrical resynchronisation than is typically achieved using echo-guided programming optimisation. Our results also indicate that optimal electrical resynchronisation is associated with the best cardiac output.

### 4.5. Limitations

The main limitation of our study is the relatively small cohort of patients included at a single centre. Risk factors and multifactorial diseases affect clinical response to CRT [26, 27] and these have not been characterised within our cohort. Long-term response to CRT is a critical outcome measure when evaluating this population; however, this study was designed to assess acute changes in ventricular performance following programming optimisation.

Whilst improvements in AHR, measured using *D*_p_/*D*_tmax_, have previously been correlated with enhanced long term response [9] this measurement technique relies upon the use of invasive haemodynamic data which did not form part of this study protocol. As such, our findings would need to be corroborated in a larger, randomised analysis before altering practice. A further limitation was the fact that this study did not address the position of the implanted LV lead used to provide LV stimulation.

No significant difference was observed in TVaT and VaT_10-90_ activation times amongst both echocardiographically and electrically optimised patients. One explanation could be the degree of scar or fibrosis present in our cohort. Since these patients did not have late enhancement CMR, the level of scarring and myocardial fibrosis is unknown. Additionally, the sensitivity of ECM, which measures epicardial activation times, to identify small, potentially intramural, late activating regions may be much less than invasive electroanatomical mapping studies. Finally, it is not possible to analyse septal depolarisation as this is not observed during epicardial mapping.

The study only considered a single acute measure, either ECM or echocardiogram to optimise device timings. Novel blood biomarkers are potential diagnostic and prognostic markers in an acute heart failure setting [28–30]. Extending our study beyond electrical and mechanical measures of cardiac function to include blood biomarkers [31–36] may further improve device setting optimisation. However, how best to integrate real time feedback from ECM and echocardiogram markers with the inherent delay in blood biomarker readings will need to be addressed.

## 5. Conclusions

Echocardiographic programming optimisation does not result in the fastest possible biventricular activation. Instead, activation was consistently more rapid when optimised via ECM than with echocardiographic optimisation. ECM-guided programming optimisation may achieve superior fusion of activation wave fronts leading to improvements in CRT response.

## Figures and Tables

**Table 1:** Patient characteristics.

Patient characteristics	Value (%)
Age (y)	69 ± 12
Sex	
** **Male	17 (81)
** **Female	4 (19)
Sinus rhythm	14 (67)
Atrial fibrillation	7 (33)
Aetiology	
** **Ischaemic	13 (62)
** **Nonischaemic	8 (38)
LV ejection fraction (%)	27 ± 10
QRS duration (ms)	162 ± 21
** **120–150 ms	6 (29)
** **>150 ms	15 (71)
QRS morphology	
** **LBBB	15 (71)
** **Non-LBBB	6 (29)

Values represent means ± SD, with percentages in parentheses where relevant. LBBB, left bundle branch block; LV, left ventricle; non-LBBB, non-left bundle branch block; RV, right ventricle.

**Table 2:** Baseline ventricular activation times.

	LVOT VTI (cm)	VaT_10-90_ (ms)	TVaT (ms)
Aetiology			
** **Ischaemic	13 ± 6	82.6 ± 5	145 ± 6
** **Nonischaemic	18 ± 6	77.6 ± 10	141 ± 9

QRS morphology			
** **LBBB	14 ± 6	84 ± 22	146 ± 26
** **Non-LBBB	17 ± 5	71 ± 10	137 ± 9

QRS duration			
** **120 to 150 ms	14 ± 7	67 ± 19	129 ± 30
** **>150 ms	16 ± 6	87 ± 19	150 ± 5

Values represent means ± SD. LBBB, left bundle branch block; LV, left ventricle; LVOT VTI, left ventricular outflow tract velocity time integral; non-LBBB, non-left bundle branch block; RV, right ventricle; TVaT, Total ventricular activation time; VaT_10-90_, ventricular activation time_10-90_. Values were compared by Mann–Whitney *U* test.

**Table 3:** Ventricular activation times acquired under the different AVD values tested.

	AVD (ms)	VaT10-90 index (ms)	TVaT (ms)
	200	81 ± 21	139 ± 24
	180	69 ± 21	136 ± 24
	160	73 ± 21	141 ± 23
	140	68 ± 19	141 ± 30
	130	66 ± 21	133 ± 29
	120	65 ± 21	135 ± 29
	110	67 ± 23	138 ± 29
	100	62 ± 20	135 ± 30
	80	64 ± 18	141 ± 24
	60	67 ± 19	147 ± 26
AVD echo-optimal (ms)		62 ± 27	139 ± 37
AVD ECM optimal (ms)		49 ± 24	111 ± 23

Values represent means ± SD. AVD, atrioventricular delay; echo, echocardiographic mapping; ECM, electrocardiographic mapping; TVaT, total ventricular activation Time; VaT_10-90_, ventricular activation time_10-90_. Values were compared by ANOVA and the Kruskal–Wallis test. No between-group comparisons were significant (Tukey's HSD).

**Table 4:** Effects of VV intervals on mean ventricular parameters.

	LVOT VTI (cm)	VaT_10-90_ index (ms)	TVaT (ms)
LV40	14.5 ± 5	65.3 ± 21	141.6 ± 28
LV30	16.1 ± 6	63.1 ± 19	140.3 ± 26
LV20	14.3 ± 5	65.8 ± 21	140.5 ± 29
LV15	16.6 ± 9	63.4 ± 19	132.0 ± 25
SIM	15.0 ± 6	68.1 ± 19	138.1 ± 25
RV20	14.8 ± 6	66.5 ± 17	135.1 ± 26
RV40	14.6 ± 6	67.3 ± 14	136.6 ± 21

LVOT VTI, left ventricular outflow tract velocity time integral; LVx, intraventricular pacing interval with the left ventricle paced first by x milliseconds; RVx, intraventricular pacing interval with the right ventricle paced first by x milliseconds; SIM, intraventricular pacing interval with the right and left ventricles paced simultaneously; TVaT, total ventricular activation time; VaT_10-90_, ventricular activation time_10-90_. Values were compared by ANOVA and the Kruskal–Wallis test. No between-group comparisons were significant (Tukey's HSD).

## Data Availability

The data used to support the findings of this study are included within the article.
