# Erratum to “Comparison of Echocardiographic and Electrocardiographic Mapping for Cardiac Resynchronisation Therapy Optimisation”

**DOI:** 10.1155/2019/3849547

**Published:** 2019-08-28

**Authors:** Helder Pereira, Tom A. Jackson, Simon Claridge, Jonathan M. Behar, Cheng Yao, Benjamin Sieniewicz, Justin Gould, Bradley Porter, Baldeep Sidhu, Jaswinder Gill, Steven Niederer, Christopher A. Rinaldi

**Affiliations:** ^1^Division of Imaging Sciences and Biomedical Engineering, King's College London, London, UK; ^2^Cardiac Rhythm Management Service, St George's University Hospitals NHS Foundation Trust, London, UK; ^3^Cardiovascular Department, Guy's and St Thomas' NHS Foundation Trust, London, UK; ^4^Medtronic Ltd, London, UK

In the article titled “Comparison of Echocardiographic and Electrocardiographic Mapping for Cardiac Resynchronisation Therapy Optimisation” [1], there was an error in Figure 1, where Figures 1(a) and 1(b) were duplicated, due to a production error. The corrected figure is shown below.

## Figures and Tables

**Figure 1:**
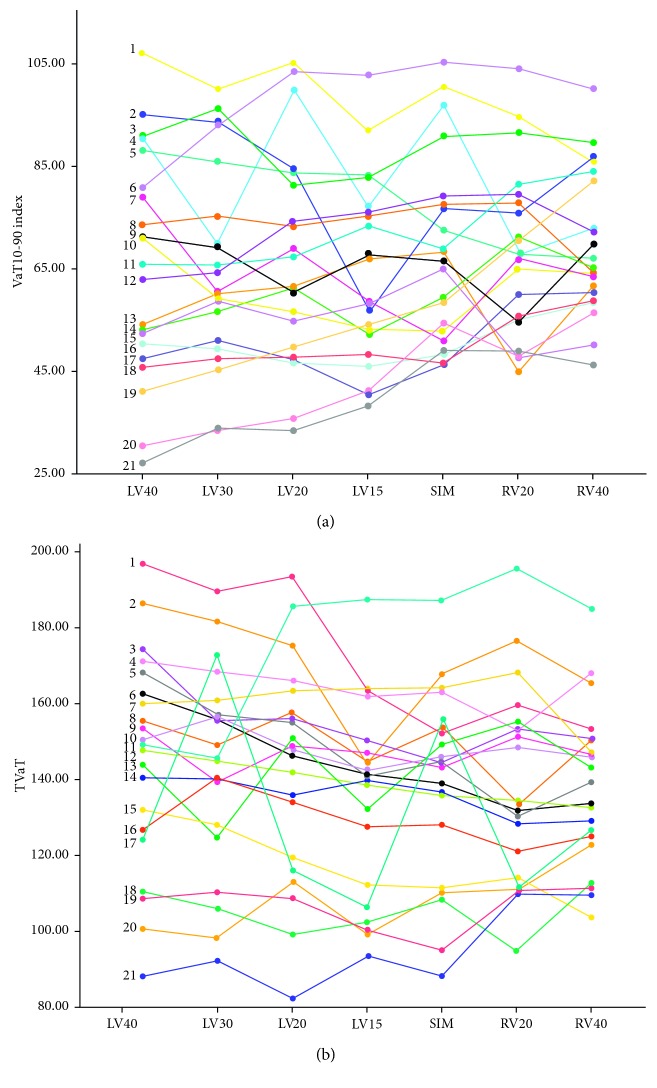
VaT10-90 and TVaT values in each patient according to the VVD setting. LVx, intraventricular pacing interval with the left ventricle paced first by *x* milliseconds; RVx, intraventricular pacing interval with the right ventricle paced first by *x* milliseconds; SIM, intraventricular pacing interval with the right and left ventricles paced simultaneously; TVaT, total ventricular activation time; VaT10-90, ventricular activation time 10–90.
